# Cross-Level Influence of Empowering Leadership on Constructive Deviance: The Different Roles of Organization-Based Self-Esteem and Traditionality

**DOI:** 10.3389/fpsyg.2021.810107

**Published:** 2022-02-02

**Authors:** Yanzi Wang

**Affiliations:** School of Economics and Management, Shanxi University, Taiyuan, China

**Keywords:** empowering leadership, organization-based self-esteem, traditionality, constructive deviance, conservation of resources theory

## Abstract

At present, scholars have mainly focused on the individual-level influencing factors of constructive deviance, and few studies have concerned the motivating mechanism of empowering leadership on constructive deviance. Based on the conservation of resources theory, this study explored the cross-level influence of empowering leadership on constructive deviance in the Chinese cultural context. With the data of 85 leaders and 383 paired employees which were collected in two waves with one-month time lag, the results demonstrated that empowering leadership motivated employees to actively implement constructive deviance, and that organization-based self-esteem played a mediating role in the relationship. The high traditionality of employees weakened not only the positive effect of organization-based self-esteem on constructive deviance, but also the mediating role of organization-based self-esteem. This study lays a theoretical basis and provides some practical guidance for leaders to take effective empowerment strategies to motivate employees to engage in constructive deviance.

## Introduction

In the VUCA era, leaders can hardly cope with the fast-changing external environment by themselves due to the limitation in management capacity and energy, such that they need to alter the traditional hierarchical management mode. Leaders should decentralize power to employees, encourage them to participate in decision-making, and share information with them in order to improve organizational flexibility through empowering behaviors (Kim and Beehr, 2020). At the same time, leaders' empowering behaviors also meet employees' psychological needs for more autonomy and give employees more chance of exerting their potentials at work (Lee et al., 2018).

Constructive deviance is defined as employees' voluntary acts that challenge important norms of the organization for higher well-being of the organization and/or its members (Galperin, 2012). In the study of Galperin (2012), constructive deviance is regarded as an independent construct. However, other scholars define constructive deviance as an umbrella term, including voice, job crafting, taking charge, extra-role behaviors, prosocial behaviors, etc (Vadera et al., 2013). At this time, constructive deviance shares definitional similarities with other pro-organizational behaviors. The key differentiation lies in that only behavior that is at the same time (1) deviant, (2) producing beneficial outcomes, and (3) conformant with hypernorms can describe constructive deviance. An influential study suggests that employee empowerment leads to constructive deviance, and that empowerment can be stimulated by transformational leadership (Vadera et al., 2013). Other leadership behaviors that have been related to employee empowerment are empowering leadership behaviors (Mertens and Recker, 2020). At present, still less is known about how empowering leadership can motivate employees to engage in constructive deviance. Many studies have shown that empowering leadership improves employees' positive work behaviors, such as voice (Jada and Mukhopadhyay, 2019), knowledge sharing behavior (Wu and Lee, 2017), innovative behavior (Amundsen and Martinsen, 2015) and organizational citizenship behavior (Bester et al., 2015). Since constructive deviance runs counter to organizational rules and challenges the authority and status of leaders, employees without essential resources support may choose not to actively implement such a behavior, according to the conservation of resources (COR) theory (Hobfoll, 1989). Empowering leadership not only provides more instrumental resources support for employees but also gives them more psychological resources support, in which case they are more likely to implement constructive deviance (Mertens and Recker, 2020). Therefore, based on the COR theory, this study explores the relationship between empowering leadership and constructive deviance.

Leaders' empowering behaviors reflect their recognition and trust in employees' abilities, which is conducive to enhancing employees' organization-based self-esteem (OBSE) (Kim and Beehr, 2018). Employees with high OBSE can create more positive psychological resources, thinking that they have the responsibility to engage actively in constructive deviance for organizational development (Zhang and Liu, 2019). However, empirical research remains scarce on the mediating role of OBSE in the relationship between empowering leadership and constructive deviance. Therefore, based on the COR theory, OBSE is used as a mediating variable to uncover the “the black box” of how empowering leadership influences constructive deviance.

**Figure 1:**
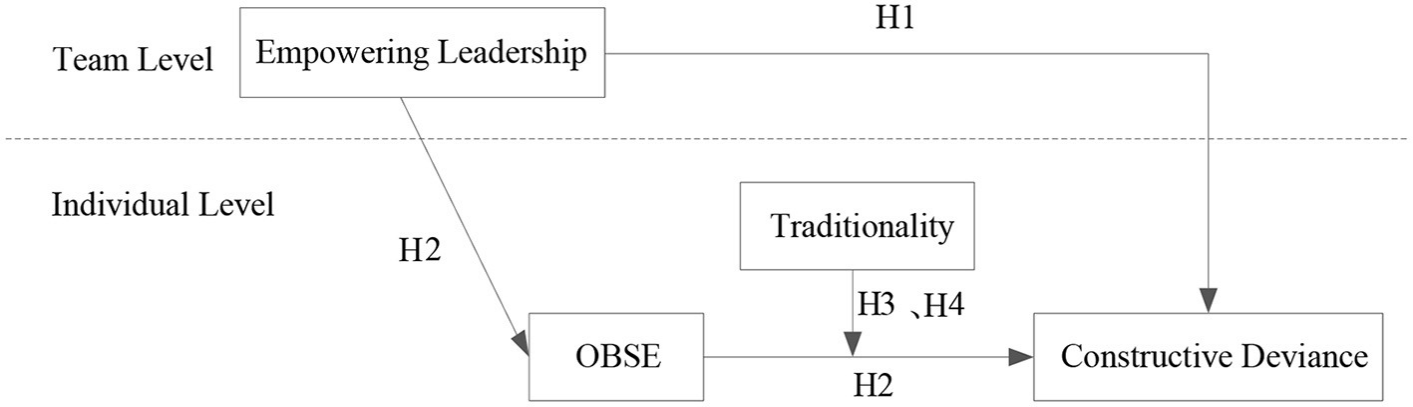
Theoretical model.

China has been affected by Confucian culture for a long time, and the deep-rooted traditional value in the Chinese Confucian culture has been ingrained in domestic corporate cultures. Chinese traditionality refers to an individual's endorsement of hierarchical role relationships as defined by the five cardinal relationships (called wu lun) in Confucianism (Yang et al., 1989). Along with the deepening of China's reform and opening up programs, traditionality as a personal disposition may vary among individuals in the Chinese context (Farh et al., 1997, 2007). Individuals with high traditionality pay more attention to fulfilling their expectations and responsibilities defined by their prescribed social roles (Li et al., 2017). On the contrary, individuals with low traditionality are oriented toward egalitarianism, self-reliance, and openness (Hu et al., 2019). Considering individual differences in value, traditionality has been introduced into the field of organizational behavior research concerning its moderating effect (Farh et al., 1997). It is believed that employees with high traditionality have high safety demand orientation and low willingness to take risks. According to the COR theory, they tend to take conservative actions to economize resources and avoid falling into the resources loss spiral (Hobfoll, 2011). They believe that constructive deviance is highly risky, and that it will run out their limited work resources. Therefore, even if OBSE can promote employees' constructive deviance, however, high traditionality could counterbalance this positive effect and thus may weaken the mediating effect of OBSE. Therefore, this study finally explores the moderating role of traditionality in the relationship between OBSE and constructive deviance and in the mediation effect of OBSE in the Chinese cultural context. The theoretical model is shown in Figure 1.

## Literature Review and Hypotheses

### Empowering Leadership and Constructive Deviance

Empowering leadership is defined as a collection of behaviors of the leader who shares power with his or her subordinates by delegating power, promoting participation in decision-making, providing guidance and information, and expressing confidence in high performance (Sharma and Kirkman, 2015). In the organization, the leader makes decisions on important matters such as performance evaluation, promotion and reward, which influences the benefit perception of employees. Therefore, the leader is the most direct and obvious factor in organizational environment that affects employees' work behaviors.

When employees perceive the threat of resources depletion or the inability of making up for depleted resources, they are more inclined to make risk-avoidance choices (Hobfoll, 1989). Suspected of violating organizational rules, constructive deviance is likely to confront employees with punishment or criticism by their leaders (Vadera et al., 2013). Therefore, the resources support from leaders can increase the success rate of constructive deviance. Empowering leaders provide employees with more access to valuable and vast work resources (such as information, guidance and performance feedback). The instrumental resources support provided by empowering leaders is conducive to reducing the threat of resources depletion perceived by employees (Kim et al., 2018), thereby motivating them to implement constructive deviance.

In addition, it is an important psychological cognitive process to weigh the potential resources gain and loss of the behavior (Halbesleben et al., 2014). Empowering leaders create a free, flexible and inclusive working atmosphere for employees to take the initiative to communicate with leaders or colleagues and learn new knowledge and skills (Jada and Mukhopadhyay, 2019). At this time, they may have less cognitive pressure and less emotional exhaustion (Lin et al., 2016). Therefore, empowering leaders can not only give employees more work-related instrumental resources support but also promote their positive psychological resources against challenges and difficulties (Lee et al., 2018). Those positive psychological resources can help employees generate more constructive deviance beyond their duties without worrying about the unpredictability and risks associated therewith (Tuckey et al., 2012). Accordingly, the first hypothesis is made as follows:

*H1:* Empowering leadership is positively related to constructive deviance.

### The Mediating Role of OBSE

OBSE is defined as employees' perception of their own importance to the organization (Pierce et al., 1989). Leaders' empowering behaviors can significantly improve employees' perception of being valuable members in the organization, thus motivate them to produce high level of OBSE (Kim and Beehr, 2018). Firstly, empowering leaders give employees high job autonomy, which means they hold part of leaders' responsibility and have more opportunities to show their own ability and make great contribution to the organization. At this time, they may experience high perception of self-efficacy, job control and job meaningfulness (Lee et al., 2017a; Hao et al., 2018), which helps them create positive psychological resources and perceive that they are of important value to the organization, and which thus generates high OBSE (Kim and Beehr, 2018). Secondly, empowering leaders are willing to hearken employees' innovative ideas and encourage them to participate actively in decision-making, which reflects leaders' trust and recognition in employees' abilities (Lee et al., 2018), and upgrades employees' positive psychological resources such as inner pleasure working in the organization. As employees are encouraged to perceive the important value of their own suggestions and innovative ideas for the development of the organization, the level of employees' OBSE is improved (Liu et al., 2013). Finally, empowering leaders provide professional guidance and feedback for employees, share information with them in time, and help them remove work obstacles. As a consequence, employees' instrumental work resources are upgraded, and employees foster a feeling that they are the key objects to be cultivated and developed by leaders (Park et al., 2017); further, employees are more likely to evaluate their personal value to the organization in a positive manner, and thus high OBSE is generated.

The COR theory points out that individuals with more resources are more likely to exhibit pro-organizational behaviors at work (Halbesleben et al., 2014). Employees with high OBSE believe that personal interests are inseparable from organizational interests. Even if their organization does not give explicit incentives for their positive work behaviors, they still have strong intrinsic motivation to take the initiative to implement risky constructive deviance (Pan et al., 2014). Firstly, employees with high OBSE tend to have more perception of work meaningfulness. These positive psychological resources improve employees' sense of organizational responsibility and prompt them to pay more attention to organizational development (Bowling et al., 2010; Deng et al., 2020). At this time, employees will engage actively in constructive deviance to contribute to the organization. Secondly, employees with high OBSE tend to believe that they are more competent than average colleagues. Based on their intrinsic motivation to maintain the consistency of self-evaluation, they tend to internalize organizational goals into their personal work goals so as to actively explore creative solutions to problems (Gardner and Pierce, 2016). Such an enrichment in positive psychological resources makes employees more energetic in the organization (Chan et al., 2013) and sets their mind free from rules. They are more willing to deviate from the norms of relevant interest groups for the sake of organizational development and engage in constructive deviance beyond regulations on their own role with a more confident attitude (Dahling and Gutworth, 2017). Accordingly, the second hypothesis is made as follows:

*H2:* OBSE mediates the relationship between empowering leadership and constructive deviance.

### The Moderating Role of Traditionality

Yang et al. (1989) defined traditionality as the extent to which a person adheres to traditional Chinese values. Traditionality was initially studied in Chinese cultural terms, and subsequently found by scholars to best characterize employees' value orientation and was thus introduced into the field of organizational behavior research (Farh et al., 1997). Employees holding different value orientations tend to differ in their sensitivity to the uncertainty (Wang et al., 2014), which may lead to significant differences in the motivating mechanism of constructive deviance. By introducing traditionality (deemed as an individual value) into the research model, this study attempts to reveal the moderating effect of traditionality in the Chinese cultural context.

Individuals with limited resources are forced to make decisions on resources allocation, and individual cognitive differences have an impact on the evaluation and allocation of their resources (Hobfoll, 2011). Firstly, employees with high traditionality tend to believe that they have inferior status in the organization (Zhang et al., 2014) so that they can't gain more work-ralated resources. Due to resources constraint, they lack self-confidence and motivation in solving work-related problems. Even if having high OBSE, they tend to pursue self-interest and security, while avoiding uncertainty as far as possible (Lu et al., 2017), so they are reluctant to carry out risky constructive deviance. Secondly, employees with high traditionality abide by their in-role obligations and focus on performing the task within the duties, and they are typically content with existing things and unwilling to accept new things, which greatly limits their divergent thinking (Wu et al., 2018). They are used to fixed ways of problem-solving and show more conservative in terms of breaking the inappropriate organizational rules which is believed to increase the chance of resources loss (Zhao, 2014). Finally, traditional Chinese culture advocates interpersonal harmony, so openly discussing organizational problems is often considered as an offense to the organization and may discomfort leaders or colleagues (Lin et al., 2016). Employees with high traditionality may think that constructive deviance breaks the fixed the work mode, threatens the interests of leaders or colleagues, and undermines the harmonious atmosphere within the organization, so they would hesitate to engage in constructive deviance that may bring them troubles or harms. They would rather maintain a friendly relationship with leaders or colleagues, avoid the interpersonal conflicts, and decrease the confrontations between individuals and organizations as far as possible (Xiang et al., 2019). Therefore, although OBSE helps to promote constructive deviance, employees with high traditionality typically regard it customary to pursue interpersonal harmony, which can weaken the positive effect of OBSE on constructive deviance.

On the contrary, employees with low traditionality are often skeptical of the existing social norms and dare to raise objections to the problems existing in the organization (Li et al., 2014). They are independent, confident, open-minded, and less concerned about the negative impact of extra-role behaviors on interpersonal relationship (Li et al., 2017). As employees with low traditionality like challenging goals and seek continuous improvement, they can always find ways to implement innovative ideas at lower risks. Even in case of low OBSE, they also dare to follow their true thoughts (Hu et al., 2019) and implement constructive deviance in breach of the unreasonable organizational rules and procedures to facilitate the development of the organization. Accordingly, the third hypothesis is made as follows:

*H3*: Traditionality negatively moderates the relationship between OBSE and constructive deviance, such that the relationship is stronger when traditionality is low rather than high.

### The Moderated Mediation Model

Based on Hypotheses 2 and 3, traditionality is assumed to also negatively moderate the mediating effect of OBSE. Individuals with limited resources are more vulnerable to resources loss and are less motivated to acquire new resources. At this time, the resources possessed by them form a vicious spiral of reduction (Hobfoll, 2011). With the belief deep-rooted in the mind of employees with high traditionality that they are lacking in sufficient instrumental resources, they doubt whether their effort will pay off, thinking that extra-role behaviors are beyond their job duties and may even ruin their existing resources (Farh et al., 2007). Therefore, even if empowering leadership improves the level of employees' OBSE, the employees with high traditionality are only willing to perform in-role duties and reluctant to spend extra time and energy engaging in constructive deviance beneficial to the organization in order to preserve existing resources (Halbesleben et al., 2014). In other words, high traditionality weakens positive psychological cognition and strengthens conservative behaviors, that is, high traditionality weakens the mediating effect of OBSE between empowering leadership and constructive deviance. Accordingly, the fourth hypothesis is made as follows:

*H4*: Traditionality negatively moderates the mediating effect of OBSE. Specifically, OBSE mediates the indirect effect of empowering leadership on constructive deviance when traditionality is low rather than high.

## Methods

### Data Collection and Sample

Survey data were collected from supervisors and their direct subordinates in 18 high-tech companies across China. Within each company, we selected a contact person through personal relationship, to whom the research objectives and attention points in the survey were clarified beforehand. The contact person was responsible for issuing and collecting the questionnaires in both supervisor's and subordinate's editions in order to keep better track of the whole investigative process. To minimize common method bias as much as possible, we collected survey data from two evaluation objects (supervisors and subordinates), over two different time periods at a one-month interval. At time 1 (T1), 500 subordinates were asked to assess empowering leadership, OBSE, and traditionality. After the questionnaires that were answered half-heartedly were ruled out, 426 questionnaires were retained. At time 2 (T2, at a one-month interval), 100 sets of corresponding supervisor's edition of questionnaires were issued, with supervisors asked to assess the constructive deviance of their direct subordinates. The initials plus the last four digits of the mobile phone number of the supervisors and their direct subordinates in each questionnaire were used as the matching method. The data of 85 supervisors and 383 subordinates were successfully matched from the two points-in-time, resulting in the final effective matching rate of 1: 4.5.

Among the supervisors, 74.1% were male; 23.5% were aged 35 years old or below, 56.4% aged between 36 and 45 years old, and 20.1% aged 46 years old or above; 9.4% had a college degree or below, 65.9% had a bachelor's degree, and 24.7% had a master's degree or above. Among the subordinates, 62.1% were male; 34.9% were aged 30 years old or below, 52.2% aged between 31 and 40 years old, and 12.9% aged 41 years old or above; most of them (71.1%) had a bachelor's degree, 15.1% had a college degree or below, and 13.8% had a master's degree or above.

### Measures

We adopted a standard back-translation procedure to translate the English scales into Chinese counterparts, aiming to ensure the equivalence of scales in both English and Chinese versions. All items were measured on five-point Likert scales ranging from 1 (strongly disagree) to 5 (strongly agree).

### Empowering Leadership

Empowering leadership was measured using 7 items from Vecchio et al. (2010). One sample item was “My supervisor urges me to think of problems as opportunities rather than obstacles.” Cronbach's α was 0.891. In this study, empowering leadership was conceptualized at the team level but assessed by subordinates, so the rationality of data aggregation needed to be tested. According to the results of data aggregation testing for empowering leadership, the average Rwg was 0.907 (> 0.7), ICC (1) was 0.398 (> 0.05), and ICC(2) was 0.725 (> 0.5), all of which met the data aggregation criteria, meaning that data aggregation was feasible.

### OBSE

OBSE was measured using 10 items developed by Pierce et al. (1989). One sample item was “I am important in the organization.” Cronbach's α was 0.867.

### Traditionality

Traditionality was measured using 5 items from Farh et al. (1997). One sample item was “When people are in dispute, they should ask the most senior person to decide who is right.” Cronbach's α was 0.878.

### Constructive Deviance

Constructive deviance was measured using 9 items from Galperin (2012), including two dimensions of organizational constructive deviance (5 items) and interpersonal constructive deviance (4 items). One sample item was “My subordinate doesn't follow my orders in order to improve work procedures.” Cronbach's α was 0.855.

### Control Variables

Following the prior studies, we controlled for the potential influences of gender (1 = male; 2 = female), educational background (1 = college degree or below; 2 = bachelor's degree; 3 = master's degree or above), and ages of the supervisors and their direct subordinates. Considering the age of supervisors is older than that of subordinates in most cases, we made distinct interval settings when counting their ages. Specifically, age was also measured as an ordinal variable (1 = 30 years old or below for subordinates/35 years old or below for supervisors; 2 = 31 to 40 years old for subordinates/36 to 45 years old for supervisors; 3 = 41 years old or above for subordinates/46 years old or above for supervisors).

## Results

### Confirmatory Factor Analysis

We used MPLUS 7.0 software to conduct a confirmatory factor analysis to evaluate the discriminant validity of the four constructs. Compared with the other alternative models (see Table 1), the four-factor model fitted the data well (χ^2^/df = 2.963, RMSEA = 0.048, SRMR = 0.056, TLI = 0.946, CFI = 0.953), thereby demonstrating the four constructs had good discriminant validity.

**Table 1:** Results of confirmatory factor analysis.

**Models**	**χ^2^/df**	**RMSEA**	**SRMR**	**TLI**	**CFI**
One-factor (EL+OBSE+T+CD)	13.451	0.193	0.209	0.334	0.352
Two-factor (EL; OBSE+T+CD)	8.145	0.142	0.172	0.642	0.631
Three-factor (EL; OBSE+T; CD)	6.723	0.124	0.142	0.765	0.746
Four-factor (EL; OBSE; T; CD)	2.963	0.048	0.056	0.946	0.953

*EL, empowering leadership; T, traditionality; CD, constructive deviance*.

### Common Method Bias Testing

Harman's single-factor test was conducted to check the common method bias. The results showed that the total variance of all factors was 81.1%, while the variance of the factor with the largest eigenvalue was 29.7%, less than 40% of the total variance, indicating that the common method bias was not rigorous.

In addition, the potential error variable control method was used to further test the common method bias. On the basis of the four-factor model, the common method bias (CMB) factor was added to construct the five-factor model in which the fit indices were χ^2^/df = 2.957, RMSEA = 0.044, SRMR = 0.053, TLI = 0.947, CFI = 0.953. Compared with the four-factor model in Table 1, the fit indices of the five-factor model had not improved significantly, indicating that the common method bias was not rigorous again.

### Descriptive Statistics and Correlations

As shown in Table 2, OBSE was positively correlated to constructive deviance (*r* = 0.378, *p* < 0.01), while traditionality was negatively correlated to constructive deviance (*r* = −0.292, *p* < 0.01).

**Table 2:** Means, standard deviations and correlations.

**Variables**	**M**	**SD**	**1**	**2**	**3**	**4**	**5**
**Individual level**							
1. Employees' gender	1.379	0.423					
2. Employees' age	1.778	0.569	0.043				
3. Employees' education	1.987	0.726	0.025	−0.078			
4. OBSE	3.352	0.672	0.053	0.041	0.072		
5. Traditionality	3.721	0.821	0.032	−0.076	0.013	0.089	
6. Constructive deviance	3.368	0.824	−0.082	−0.046	0.025	0.378**	−0.292**
**Team level**							
1. Leaders' gender	1.259	0.321					
2. Leaders' age	1.965	0.478	0.035				
3. Leaders' education	2.153	0.523	0.027	0.038			
4. Empowering leadership	4.021	0.396	0.056	0.057	0.068		

*Individual level N = 383; Team level N = 85*;

** *p < 0.01 (two-tailed)*.

### Hypothesis Testing

Considering the nested data structure in this study, it was necessary to use a cross-level model to test the foregoing hypotheses. A null model with no predictors was built to calculate the intragroup variance (σ^2^= 0.213), intergroup variance (τ_00_ = 0.365), and ICC value (0.631) of constructive deviance by MPLUS 7.0 software. The ICC value was much higher than the cutoff value of 0.059, indicating that constructive deviance had great proportion of variance at the team level. Accordingly, the data were suitable for the cross-level analysis.

#### Main and Mediating Effect Testing

MPLUS 7.0 software was employed to test the main and mediating effects. All variables were mean-centered to ease the multicollinearity before the hierarchical regression analysis was conducted. As shown in Table 3, empowering leadership had a significantly positive effect on constructive deviance (Model 2, γ_01_ = 0.418, *p* < 0.01), so Hypothesis 1 was supported. The regression coefficient of empowering leadership on constructive deviance was smaller but still significant (Model 4, γ_01_= 0.276, *p* < 0.01) when both empowering leadership and OBSE were introduced into the regression equation, indicating OBSE played a partial mediating role. The result lent a support to Hypothesis 2.

**Table 3:** Results of hierachical regression analysis.

**Variables**	**Constructive deviance**					
	**Model 1**	**Model 2**	**Model 3**	**Model 4**	**Model 5**	**Model 6**
Employees' gender	−0.056	−0.043	−0.042	−0.028	−0.040	−0.032
Employees' age	−0.023	−0.029	−0.021	−0.007	−0.023	−0.013
Employees' education	0.016	0.011	0.015	0.011	0.010	0.003
Leaders' gender	−0.013	−0.021	−0.016	−0.003	−0.011	−0.008
Leaders' age	0.010	0.009	0.005	0.008	0.003	0.006
Leaders' education	0.011	0.013	0.006	0.003	0.004	0.005
Empowering leadership (*γ_01_*)		0.418**		0.276**		
OBSE (*γ_10_*)			0.325**	0.248**	0.301**	0.252**
Traditionality (*γ_20_*)					−0.258**	−0.202**
OBSE × Traditionality (*γ_30_*)						−0.163*
Intercept (*γ_00_*)	2.486*	2.112*	3.162**	2.006*	3.365**	3.672**
Intragroup variance (σ^2^)	0.068	0.158	0.163	0.123	0.173	0.189
Intergroup variance (*τ_00_*)	0.035	0.463	0.324	0.407	0.356	0.435

* *p < 0.05*;

** *p < 0.01 (two-tailed)*.

In addition, the robustness of the mediating effect was tested by bootstrapping 10,000 samples using R software. The resampling-based bootstrapping method of MPLUS 7.0 software is not suitable for the cross-level data in estimating the confidence interval (CI), so the parameter-based bootstrapping method of Monte Carlo simulation in the R software was employed to estimate the confidence interval. Consistent with Hypothesis 2, the indirect effect of empowering leadership on constructive deviance via OBSE was significant (β = 0.185, 95% CI [0.092, 0.273], excluding 0), and the direct effect was also significant (β = 0.211, 95% CI [0.065, 0.315], excluding 0), indicating that OBSE played a partial mediating role again.

#### Moderating Effect Testing

Likewise, MPLUS 7.0 software was used to test the moderating effect of traditionality. As shown in Table 3, the interaction coefficient between OBSE and traditionality was negative and significant (Model 6, γ_30_ = −0.163, *p* < 0.05), indicating traditionality played a negative moderating role in the relationship between OBSE and constructive deviance. Therefore, Hypothesis 3 was supported.

**Figure 2:**
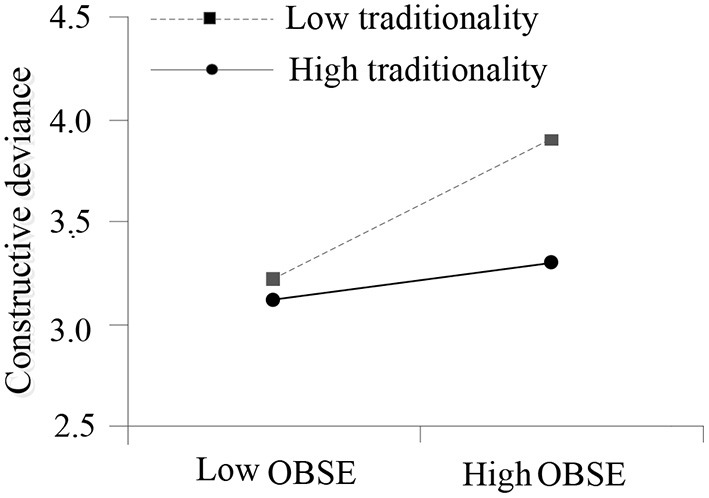
Interaction effect of OBSE and traditionality on constructive deviance.

Although the core variables involved in the moderating effect testing were at individual level, the control variables such as leaders' gender, age and education in Table 3 were at team level. Similar to the robustness testing on the mediating effect of OBSE, a more rigorous testing was conducted on the moderating effect of traditionality using R software based on the parameters calculated by MPLUS 7.0 software. The effect of OBSE on constructive deviance was not significant (β = 0.073, 95% CI [−0.079, 0.321], including 0) when traditionality was high (one standard deviation above the mean), but was significant (β = 0.388, 95% CI [0.102, 0.345], excluding 0) when traditionality was low (one standard deviation below the mean). Moreover, the value of difference in moderating effect between high and low levels of traditionality was also significant (β = −0.315, 95% CI [−0.267, −0.102], excluding 0). Addtionally, simple slope analysis was used to further explore this interaction effect. As shown in Figure 2, the positive relationship between OBSE and constructive deviance was stronger when traditionality was low rather than high, that is, high traditionality greatly weakened the positive relationship beweeen OBSE and constructive deviance. The result lent further a support to Hypothesis 3.

#### Moderated Mediating Effect Testing

Mplus7.0 and R softwares were used to test the moderated mediating effect. Mplus7.0 software was used to calculate the conditional indirect effect coefficient, and then R software was used to estimate the confidence interval. As shown in Table 4, the mediating effect of OBSE in the relationship between empowering leadership and constructive deviance was no longer significant (β = 0.025, 95% CI [−0.043, 0.245], including 0) at a high level of traditionality, but it remaind significant (β = 0.186, 95% CI [0.074, 0.286], excluding 0) at a low level of traditionality. Moreover, the value of difference in conditional indirect effect between high and low levels of traditionality was significant (β = −0.161, 95% CI [−0.203, −0.089], excluding 0). The result indicated that the moderated mediating effect was significant, and Hypothesis 4 was supported.

**Table 4:** Results of moderated mediating effect testing.

**Traditionality level**	**Conditional indirect effect**	**Standard error**	**95% confidence interval**	
			**The lower limit**	**The upper limit**
High (M+1SD)	0.025	0.036	−0.043	0.245
Low (M-1SD)	0.186	0.047	0.074	0.286
High-Low	−0.161	0.029	−0.203	−0.089

## Conclusions and Prospects

### Discussion

This study provided a better understanding of why empowering leadership could stimulate employees' constructive deviance. OBSE and traditionality, which were regarded as important intrinsic motivation resources, had been introduced into the research model based on the COR theory in this study. The results showed empowering leadership had a significantly positive influence on constructive deviance, and that the positive relationship was transferred via the mediating role of OBSE and was contingent upon traditionality.

The COR theory can explain why empowering leadership contributes to generating constructive deviance. It points out that the consumption of resources without corresponding supplementation will bring great stress to individuals, at which time they will engage in the activities conducive to reducing resources consumption and preserving existing resources (Hobfoll, 2011). As a trial and error behavior with certain risks, constructive deviance accelerates the consumption of the existing resources of employees, so they are reluctant to actively implement it (Kura et al., 2016). Leaders' empowering behaviors such as delegating power, providing autonomy, elevating participation in decision-making can enrich employees' work resources, enhance employees' perception of control over work, and reduce the uncertainty of outcomes of work (Kim et al., 2018). In those cases, employees can get more job security in the organization and become ready to take risks to engage in constructive deviance. This result is consistent with previous research findings that empowering leadership can enhance a series of employees' proactive behaviors. The present study further highlights the importance of empowering leadership to the organization.

Empowering leadership can inspire employees to engage in constructive deviance via the mediating role of OBSE. Leaders' empowering behaviors enhance employees' self-evaluation of individual importance to the organization, which is conductive to improving employees' OBSE (Kim and Beehr, 2018). Employees with high OBSE tend to think they have a strong sense of ownership in the organization. The positive psychological cognitive resources enhance their intrinsic motivation to initiatively implement constructive deviance (Lapointe et al., 2011). The result is consistent with the previous argument that the sense of self-worth at work (OBSE) can serve as a driver in positive work behaviors. At the same time, this study has also demonstrated the unique value of empowering leadership in increasing the intrinsic motivation resources of employees.

Traditionality can not only negatively moderate the relationship between OBSE and constructive deviance, but also the mediating effect of OBSE in the Chinese cultural context. Employees with high traditionality tend to believe that they have limited work resources, so they are only willing to conform to the role duties and obligations expected by the society, and resist change (Zhao and Liu, 2020). Therefore, they can't be motivated to implement constructive deviance by the improvement of OBSE, that is, high traditionality weakens the mediating effect of OBSE. On the contrary, employees with low traditionality are not afraid to make mistakes and dare to take risks in breach of inappropriate organizational rules without worrying about that the potential failure may cause them to suffer from the losses of image, status, and work resources (Wang et al., 2014). In other words, employees who exhibit low traditionality and high OBSE at the same time are more likely to engage in constructive deviance. These results are similar to the findings of Zhao (2014) that traditionality moderated the mediating effect of affective commitment on the relationship between RLMX and voice. Previous studies has suggested that the influence of traditionality should receive more attention in the Chinese cultural context (Li et al., 2017; Wu et al., 2018). We believe that examining the influence of traditionality at the individual level is a fruitful way of studying the effects of in dividual cultural values on behaviors. In sum, this study has undoubtedly deepened the understanding of how traditionality affects the relationship between empowering leadership and constructive deviance in the Chinese cultural context.

### Theoretical Contributions

The present study has contributed to the literatures on constructive deviance in two ways. First, this study explored the motivating mechanism of constructive deviance from the perspective of leadership. Previous studies mainly focused on the impact of individual-level factors on constructive deviance, and rarely explored the motivating factors of constructive deviance from the perspective of leadership (Mertens et al., 2016). As an effective managerial strategy to cope with the fast-changing external environment, leaders' empowering behaviors motivate employees to break the inappropriate organizational rules and further carry out constructive deviance. Based on the COR theory, this study explored the relationship between empowering leadership and constructive deviance, and widened the scope of leadership that stimulates employees' constructive deviance.

Second, this study revealed the influencing mechanism of empowering leadership on constructive deviance by allowing for the mediating role of OBSE. Few studies were focused on OBSE as a mediating variable between empowering leadership and constructive deviance (Lee et al., 2018). Based on the COR theory, this study introduced OBSE as a mediator to represent the enrichment of employees' psychological resources due to empowering leadership, aiming to expand the research on the mediating mechanism between empowering leadership and constructive deviance, thereby clarifying the “black box” of the relationship.

Third, based on the Chinese cultural context, this study provided a more nuanced comprehension in the motivational mechanism of constructive deviance by allowing for the moderating role of traditionality. Traditionality reflects the individual value difference of whether they dare or not to implement constructive deviance. Based on the COR theory, this study introduced traditionality as a moderator that reflects employees' anxiety about and fear of depletion of work resources, and then explored its boundary effect on the relationship between OBSE and constructive deviance and the moderated mediating effect, greatly enriching the existing theories on constructive deviance in the Chinese cultural context. These findings responded to discussions of cultural differences in the study of constructive deviance (Vadera et al., 2013) and provided further empirical support for exploring the impact of cultural value differences on how employees react to constructive deviance.

### Managerial Implications

This study carries several important managerial implications. First, leaders should empower their subordinates effectively to motivate them to engage in constructive deviance. Leaders should entitle employees to autonomy, encourage them to participate in decision-making, express their high performance expectations, and share important information with them so that they can access more work resources and improve their psychological safety, and take further initiative to implement constructive deviance without any worry about and fear of resources loss.

Second, leaders should promote the level of employees' OBSE through empowering behaviors. Employees' self-esteem at work may be formed through the way leaders have treated them. Leaders' empowering behaviors signal to their surbordinates that they are valuable and important members to the organization, leading to a high self-esteem at work (OBSE). Such a positive self-awareness can motivate employees to implement constructive deviance to embody their self-worth as an important member to their organization.

Third, leaders should adopt diversified management strategies specific to the different levels of employees' traditionality. For the employees with low traditionality, leaders should enhance their self-worth at work through more empowering behaviors and further motivate them to initiatively implement constructive deviance. For the employees with high traditionality, leaders should enhance their psychological safety by creating an open and fault-tolerant work atmosphere, and provide them with necessary coaching and training so as to improve their willingness and capability of implementing constructive deviance.

### Limitations and Future Research Directions

Despite the achievements made in this study, a few limitations should be noted. First, similar to most previous studies, this study also demonstrated that empowering leadership had a positive effect on employees' behaviors, but the “double-edged sword” effect of empowering leadership was overlooked in the current study. On the one hand, empowering leadership can inspire employees' intrinsic motivation; on the other hand, empowering leadership can also increase employees' work pressure, i.e., the so-called “double-edged sword” effect of empowering leadership on employees' behaviors which some scholars have believed in recently (Cheong et al., 2016). Therefore, future research remains to further explore whether empowering leadership has such a “double-edged sword” effect on constructive deviance.

Second, recent studies have shown that there is a nonlinear relationship between empowering leadership and task performance, that is, the effect of “too much of a good thing is bad” (Lee et al., 2017b; Cheong et al., 2019). The scope of empowerment seems too narrow to ensure the imbalance in rights, responsibilities and interests of employees, but that is too wide so as to go beyond their competence. Therefore, the scope of empowerment should be modest. In fact, more and more studies have shown that the non-linear effect between variables is more consistent with objective practice. Therefore, future research remains to further explore whether empowering leadership has such a non-linear effect on constructive deviance.

Third, the research model was not inclusive of all the motivational factors of constructive deviance. According to the proactive motivation model proposed by Parker et al. (2010), constructive deviance is also a kind of proactive behavior. It follows that the motivational factors (e.g. employees' regulatory focus and core self-evaluation at individual level, transformational leadership and ethical leadership at team level, organizational culture and positive organizational support at organizational level) of such a proactive behavior put forward by the proactive motivation model also apply to constructive deviance. Future research can explore the interactive mechanism among individual, team and organizational factors on constructive deviance. In addition, research in the education field suggests that cognitive maturity is positively correlated with problem-solving ability (Macpherson, 2002). Generally speaking, employees with high problem-solving ability will actively implement constructive deviance. Therefore, we can speculate the cognitive maturity of the employees encourage the constructive deviance. Future research can take cognitive maturity as a predictor or moderator to explore its effect on constructive deviance.

Lastly, although this study collected the paired data at two time points, it was still unable to accurately identify the causal relationship among variables. Therefore, future research can use cross lagged panel design to better explore the causal relationship among variables.

## Data Availability Statement

The raw data supporting the conclusions of this article will be made available by the authors, without undue reservation.

## Ethics Statement

Ethical review and approval was not required for the study on human participants in accordance with the local legislation and institutional requirements. Written informed consent for participation was not required for this study in accordance with the national legislation and the institutional requirements.

## Author Contributions

The author confirms being the sole contributor of this work and has approved it for publication.

## Funding

The work was funded by National Natural Science Foundation of China (72071124) and the Humanities and Social Science Foundation of the Ministry of Education of China (20YJC630152).

## Conflict of Interest

The author declares that the research was conducted in the absence of any commercial or financial relationships that could be construed as a potential conflict of interest.

## Publisher's Note

All claims expressed in this article are solely those of the authors and do not necessarily represent those of their affiliated organizations, or those of the publisher, the editors and the reviewers. Any product that may be evaluated in this article, or claim that may be made by its manufacturer, is not guaranteed or endorsed by the publisher.
